# Adaptive auditory risk assessment in the dogbane tiger moth when pursued by bats

**DOI:** 10.1098/rspb.2010.1488

**Published:** 2010-08-18

**Authors:** John M. Ratcliffe, James H. Fullard, Benjamin J. Arthur, Ronald R. Hoy

**Affiliations:** 1 Center for Sound Communication, Institute of Biology, University of Southern Denmark, 5230 Odense M, Denmark; 2 Department of Biology, University of Toronto at Mississauga, Ontario, CanadaL5L 1C6; 3 Department of Neurobiology and Behavior, Cornell University, Ithaca, New York, NY 14853, USA

**Keywords:** predator–prey interaction, sound-producing moths, *Cycnia tenera*, echolocating bats, acoustic aposematism, neuroethology

## Abstract

Moths and butterflies flying in search of mates risk detection by numerous aerial predators; under the cover of night, the greatest threat will often be from insectivorous bats. During such encounters, the toxic dogbane tiger moth, *Cycnia tenera* uses the received intensity, duration and emission pattern of the bat's echolocation calls to determine when, and how many, defensive ultrasonic clicks to produce in return. These clicks, which constitute an acoustic startle response, act as warning signals against bats in flight. Using an integrated test of stimulus generalization and dishabituation, here we show that *C. tenera* is able to discriminate between the echolocation calls characteristic of a bat that has only just detected it versus those of a bat actively in pursuit of it. We also show that *C. tenera* habituates more profoundly to the former stimulus train (‘early attack’) than to the latter (‘late attack’), even though it was initially equally responsive to both stimuli. Matched sensory and behavioural data indicate that reduced responsiveness reflects habituation and is not merely attributable to sensory adaptation or motor fatigue. In search of mates in the face of bats, *C. tenera*'s ability to discriminate between attacking bats representing different levels of risk, and to habituate less so to those most dangerous, should function as an adaptive cost–benefit trade-off mechanism in nature.

## 1. Introduction

The primary function of a moth's ear is to detect bat biosonar [1,2], and moth's ears have almost certainly evolved through and are maintained by selective pressures from sympatric insectivorous echolocating bats [3,4]. In response to the echolocation calls of a typical aerially hawking bat, noctuoid moths' auditory systems evoke evasive flight [1] and, in many tiger moths, also defensive sound production [5,6]. Both of these behaviours constitute acoustic startle responses (ASRs), the neural control of which has been the subject of a number of studies at both the invertebrate and vertebrate levels [7]. Over the course of a bat's attack, echolocation call rate increases while call duration decreases [8]. Early in the approach phase, bats have detected and localized their target; later in this phase bats actively plot a course for target interception [9]. Noctuid moths are thought to estimate bat predation risk using a combination of echolocation call rate and intensity as received at their two tympanal ears [10,11], each of which contains only two auditory afferents (the A1 and A2 cells; A2 being roughly 20 dB less sensitive than A1; [1]).

The dogbane tiger moth, *Cycnia tenera* is unpalatable to bats [12] and produces defensive ultrasonic clicks (i.e. phonoresponds) to the echolocation calls of bats nearby [13]. Here too a combination of received echolocation call rate and intensity elicits this anti-bat defence [6,11,13,14]. For a given duty-cycle of bat echolocation calls, or simulated bat echolocation-like sounds, the dogbane tiger moth phonoresponds preferentially to pulse repetition rates between 15 and 90 Hz [11,13,14]. This broad range corresponds to the approach phase of an aerial hawking bat's attack sequence [8,13].

A recent test of stimulus generalization between ultrasound simulating a searching bat (repetition rate = 4.65 Hz) and an attacking bat (repetition rate = 46.5 Hz) suggests that *C. tenera* recognizes a pre-detection bat as distinct from a post-detection bat and exhibits its ASR (i.e. phonoresponds) using the bat's call emission rate [11]. Given competing demands upon its severely limited time and energy budget, we assume that *C. tenera* would do well to continuously re-assess what is the tolerable level of risk exposure and reduce its phonoresponse accordingly over repeated bat encounters.

In the study we report here, we tested the hypothesis that *C. tenera* is able to discriminate between pulse trains simulating attacking bats posing different levels of risk but to which the moth is initially equally responsive. We also tested the prediction that moths would exhibit a greater decrease in responsiveness to repeated presentations of simulated early-approach phase attack calls (lower risk) than to simulated late-approach phase attack calls (higher risk), representing a simple form of iterative risk-assessment and reflecting a trade-off between reproduction and longevity. To do so, we integrated tests of stimulus generalization and dishabituation (as suggested by Wyttenbach & Hoy [15]). Pulse rates used were within the preferred range for eliciting sound production and fell on opposite sides of the rate most likely to elicit defensive clicks (approx. 45 Hz; [14]). Following behavioural assays, we made extracellular electrophysiological recordings of the moths' auditory neural activity to these same digital recordings and, for each simulated bat echolocation call (hereafter, a ‘pulse’), noted A1 and A2 auditory receptor cell activity. In doing so, we hoped to unravel the roles of sensory adaptation and habituation in the reduction in defensive sound production (i.e. responsiveness) and the relative importance of (i) individual sensory bursts, with respect to spike number and instantaneous spike period, and (ii) the repetition rate at which a series of sensory bursts occur.

## 2. Material and methods

Experiments were conducted near Chaffey's Lock, Ontario, Canada at the Queen's University Biological Station (QUBS). *Cycnia tenera* were reared from eggs collected from wild-caught females and raised to pupae on dogbane (*Apocynum androsaemifolium*) and Indian hemp (*A. cannabinum*). Pupae were over-wintered at the University of Toronto in constant temperature rooms at 4°C (12 L : 12 D regime) for several months and then transferred to constant temperature rooms at 25°C (16 L : 8 D). Adults emerged two to three weeks later at QUBS and were allowed to mature for 12–24 h before being used as subjects. Both early and late attack groups (described below) comprised 10 moths: 5 males and 5 females, for a total of 20 moths.

### (a) Behaviour

Moths were tethered from their dorsal thorax using wax and rigid wire in a dark chamber lined with sound-absorbing foam. Moths were allowed to acclimate for 20 min and remained relatively motionless throughout trials. Acoustic stimuli and tymbal clicks were detected using a condenser microphone (CM16, Avisoft, Berlin, Germany) equidistant from moth and speaker, and recorded using an acquisition board (sampling rate = 250 kHz, UltraSoundGate 416-200, Avisoft) connected to a PC running Avisoft Recorder. Digital recordings (.wav files) were subsequently analysed using BatSound Pro v. 3.2 (Pettersson Elektronik AB, Uppsala, Sweden). We noted the number of click modulation cycles (MCs; the 14–20 clicks produced over the course (approx. 20 ms) of a single tymbal's collapse and recovery) and each MC's onset time as referenced to the beginning of each trial (i.e. the time at which the first pulse reached the moth's ipsilateral ear).

### (b) Neurophysiology

Approximately 30 min after behavioural trials had been run, we prepared the same moths for extracellular electrophysiology. We used standard techniques [16] to expose the auditory nerve (IIIN1b) and recorded the action potentials from the A1 and A2 auditory receptor cells in response to acoustic stimuli with a stainless steel hook electrode referenced to another placed in the moth's abdomen. Moths were not decapitated to avoid reduction in the phonoresponse [17]. Responses were amplified (P15, Grass Instruments, Astro-Med, West Warwick, RI), digitized (sampling rate = 20 kHz, TL2, Axon Instruments, Foster City, CA USA) and stored on a PC. All records were analysed using a custom Matlab application.

We report the minimum instantaneous auditory receptor cell action potential (spike) period (minISP) rather than the average spike rate (spikes s^−1^) for sensory bursts, as minISP is a more direct measure of afferent activity [10] and probably a better predictor of postsynaptic processing [18].

### (c) Stimuli

Intact moths and auditory preparations were exposed to pulsed synthetic sounds generated by a Matlab application (v. R2006b, The MathWorks, Natick, MA, USA) running on a desktop PC, broadcast via a high-speed data acquisition card (DAQCard 6062E and DAQ USB, National Instruments, Austin, USA), ultrasonic amplifier (70 101, Avisoft Bioacoustics, Berlin, Germany) and ultrasonic speaker (ScanSpeak 60 102, Avisoft). The speaker was mounted 20 cm behind and ventral to the moth in both the chamber (§2*a*) and Faraday cage (§2*b*). Pulse intensities were recorded as voltages delivered to the speaker and then converted to peak equivalent sound pressure levels (dB peSPL; RMS re 20 µPa) from equal-amplitude continual tones measured using a measuring amplifier (model 2610, Brüel & Kjær (B&K), Nærum, Denmark) and 6.35 mm condenser microphone (model 4135, B&K) at the moths' eventual position. The entire system was calibrated throughout using a pistonphone (model 4228, B&K).

**Figure 1.**
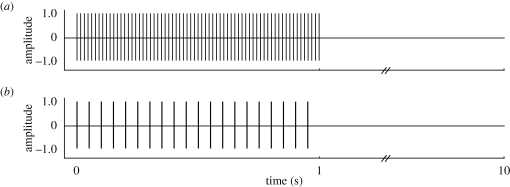
Time–amplitude traces of the two acoustic stimulus trains presented to moths. (*a*) Complete late-attack stimulus train over the course of a single trial (including 9-s of silence). This was the trial design used on trials 1–30 and 32 for the late-attack group and that used on trial 31 for the early-attack group. Each train contains sixty-seven 1.5 ms 50 kHz pulses. (*b*) Complete early-attack stimulus train (including 9 s of silence) over the course of a single trial. This was the trial design used on trials 1–30 and 32 for the early-attack group and that used on trial 31 for the late-attack group. Each train contains twenty 5 ms 50 kHz pulses (see figure 2).

‘Early-attack’ and ‘late-attack’ stimulus trains were both consistent with the rates, design and duty cycle of echolocation calls emitted by the bat species common at our study site during the approach phase of their aerial hawking attacks [8,19,20]. Specifically, early-attack stimulus trains consisted of twenty 50 kHz, 5 ms pulses (0.5 ms rise/fall time) with a pulse period of 50 ms, resulting in a duty cycle of 10 per cent and pulse rate of 20 Hz. Early-attack pulses simulate the acoustic cues of a bat that has just detected a target. Late-attack stimulus trains consisted of sixty-seven 50 kHz, 1.5 ms pulses (0.5 ms rise/fall time) with a pulse period of 15 ms, resulting in a duty cycle of 10 per cent and a pulse rate of 67 Hz. Late-attack calls simulate the acoustic cues of a bat on a collision course. The .wav files for both stimuli train designs had 9 s of silence added after the initial 1 s of pulsed sounds (figure 1). One trial was therefore 10 s in total duration, consisting of 1 s of pulsed sound (the stimulus train) followed by 9 s of silence (figure 1). All pulses were 95 dB at the moths' ears.

We note that during a real attack sequence, a bat's call rate increases continuously over the approach phase [8], and that in choosing steady rates at either end of this continuum we have sacrificed some ecological relevance so as to present the moths with two stimulus trains to which the moths were equally responsive but that reflect different levels of risk from bats to the moths. Bats reduce emitted call intensity as the distance to the target decreases and 95 dB is a reasonable sound pressure level for both early- and late-phase approach calls to arrive at the moths' ears [21].

To test the hypothesis that *C. tenera* discriminates between early- and late-approach phase echolocation calls, we used an integrated test of stimulus generalization and dishabituation to substantiate stimulus discrimination. Independently, each of these designs is an appropriate means of testing an animal's ability to discriminate between two stimuli [22]. To this end, 10 moths (early-attack phase group) were played back 30 looped repetitions of simulated early-attack phase calls (trials 1–30), then simulated late-attack phase calls (trial 31, test of stimulus generalization) and then early-attack phase calls once more (trial 32, test of dishabituation) (total duration of all 32 trials = 320 s). Another 10 moths (late-attack phase group) were presented with the same design replacing early-attack phase calls with late-attack phase calls for trials 1–30 and 32, and late-attack phase calls with early-attack phase calls on trial 31.

## 3. Results

### (a) Behaviour

The number of MCs produced in response to trial 1 did not differ significantly between moths exposed to early-attack calls and moths exposed to late-attack calls (two-sample *t*-test, *n* = 20, *p* = 0.9; figure 3). Initial response equivalency is a critical requirement for sensitivity when using a stimulus generalization paradigm [15,22], an assumption not met in [11], where initial responsiveness to searching bats was significantly lower than that of attacking bats. The phonoresponse tended to ramp up and then plateau during each stimulus train (figure 4). After pulses stopped, clicking typically continued for 0.5–1 s into the 9 s of playback silence, rarely for greater than 2 s (figure 4).

Over the first 30 trials, both early-attack and late-attack groups habituated to the stimulus train, but the early-attack group more profoundly (*y* = 36.87 − 0.66*x*, *r*^2^ = 0.88, *p* < 0.001) than did the late-attack group (*y* = 43.05 − 0.41*x*, *r*^2^ = 0.88, *p* < 0.001) (two-tailed test for difference between two population regression coefficients, *t* = 2.08, *p* = 0.04; figure 3). The magnitude of the phonoresponse in the early-attack group to the single late-attack pulse train (trial 31) was significantly greater than that of trial 30 (paired *t*-test, *n* = 10, *p* < 0.001; figure 3). The opposite relationship was significant in the late-attack group (paired *t*-test, *n* = 10, *p* = 0.02; figure 3). Therefore, moths did not generalize between early- and late-attack calls. However, neither group dishabituated to its conditioned stimulus (trial 30 MCs versus trial 32 MCs, two-paired *t*-tests, *n* = 10 for each, *p* = 0.14 for early-attack group, *p* = 0.26 for late-attack group; figures 3 and 4).

### (b) Neurophysiology

While MC number gradually decreased over trials 1 through 30 for both early- and late-attack groups (figure 3), sensory cell neural activity (A1 and A2 spike number and minISP) per pulse (i.e. simulated bat call) did not differ between trials 1, 30 and 32 in either group (eight repeated-measures analysis of variance (ANOVA); *n* = 10, *p* > 0.05 for all; table 1). Mean neural activity did not differ between groups to either the early- or late-attack stimulus trials (four two-sample *t*-tests, *n* = 20, *p* > 0.5 for all). We therefore pooled data from both groups at the individual level. For early-attack pulses, average A1 cell activity over all trials was 5.17 ± 0.81 spikes (mean ± 1 s.d.) and 1.84 ± 0.17 minISP (*n* = 20); average A2 activity was 4.24 ± 1.03 spikes and 1.95 ± 0.29 minISP (*n* = 19, one moth never exhibited A2 activity). For late-attack pulses, average A1 cell activity over all trials was 2.21 ± 0.42 spikes and 2.45 ± 0.35 minISP (*n* = 20); average A2 activity was 1.61 ± 0.47 spikes and 2.58 ± 0.36 minISP (*n* = 19, one moth never exhibited A2 activity). Average A1 and A2 activity was significantly greater (i.e. higher spike number/pulse, lower ISP) in response to individual early-attack pulses than to individual late-attack pulses (four paired *t*-tests, *n* = 20; *p* < 0.001 for all).

**Table 1.** Between- and within-trial A1 and A2 cell activity (i.e. spike number and inter-spike period). All eight repeated-measures ANOVA (between trials) were insignificant (*p* > 0.05 for all) while all eight paired *t*-tests (within trials) were significant (*p* < 0.05 for all), such that A1 and A2 cell spike number was higher in response to the first pulse than the last pulse within a trial and that A1 and A2 minISP was lower in response to the first pulse than to the last (note that the lower the ISP the faster the instantaneous spike rate). ‘Last pulse’ refers to the 20th pulse of the early-attack stimulus train and the 67th pulse of the late-attack stimulus train. Note mean A1 ISP was always less than or equal to 2.6 ms, the threshold minISP suggested as required for initiating evasive flight [1].

	mean A1 and A2 cell activity per pulse (between trials)				A1 and A2 cell activity per pulse (within trials)		
	trial 1	trial 30	trial 32		first pulse	last pulse	
	(mean ± s.d.)	(mean ± s.d.)	(mean ± s.d.)	*p*	(mean ± s.d.)	(mean ± s.d.)	*p*
*early-attack group*							
A1 spike no.	5.26 ± 0.77	5.17 ± 0.80	5.08 ± 0.88	0.22	6.72 ± 1.10	4.93 ± 0.83	<0.001
A1 minISP	1.83 ± 0.17	1.83 ± 0.19	1.86 ± 0.18	0.71	1.62 ± 0.17	1.91 ± 0.16	<0.001
A2 spike no.	4.27 ± 1.09	4.28 ± 1.02	4.19 ± 1.01	0.46	5.78 ± 1.37	3.96 ± 1.03	<0.001
A2 minISP	1.94 ± 0.31	1.93 ± 0.29	1.97 ± 0.30	0.19	1.66 ± 0.24	1.99 ± 0.37	0.002
*late-attack group*							
A1 spike no.	2.18 ± 0.36	2.20 ± 0.40	2.25 ± 0.47	0.15	4.50 ± 1.08	1.70 ± 0.67	<0.001
A1 minISP	2.43 ± 0.38	2.46 ± 0.32	2.46 ± 0.35	0.87	1.67 ± 0.23	2.32 ± 0.22	0.005
A2 spike no.	1.60 ± 0.48	1.62 ± 0.50	1.61 ± 0.43	0.51	3.70 ± 1.42	1.40 ± 0.84	<0.001
A2 minISP	2.56 ± 0.36	2.59 ± 0.35	2.59 ± 0.36	0.79	1.88 ± 0.40	2.48 ± 0.56	0.002

Average total number of A1 and A2 spikes in response to an early-attack trial was 100 and 76, respectively, to a late-attack trial, 134 and 94. Total combined A1 and A2 spike counts were significantly greater in response to late-attack trials than in response to early-attack trials (paired *t*-test, *n* = 20; *p* < 0.001).

Across trials 1, 30 and 32, auditory afferent activity levels were stable (table 1). However, sensory adaptation was observed *within* these trials with respect to A1 and A2 spike number and A1 and A2 minISP (data pooled at the individual level, pulse 1 activity versus pulse 20 activity for early-attack trials, pulse 1 activity versus pulse 67 activity for late-attack trials; eight paired *t*-tests, *n* = 20, *p* < 0.05 for all; table 1). Such sensory adaptation is consistent with previous reports [23] but is in contrast to the overall increase in behavioural responsiveness over the course of single trials, often beyond the endpoint of acoustic stimulation (figure 4). This may, in part, be explained by the fact that even during sensory adaptation, A1 and A2 minISP were typically shorter than the A1 minISP suggested by Roeder [10] as necessary for eliciting evasive flight (table 1). We note that at the individual level, in both groups of moths, the rate of within-trial sensory adaptation (i.e. decrease in spike number/pulse, increase in minISP/pulse) was remarkably similar across trials 1, 30 and 32.

**Figure 2.**
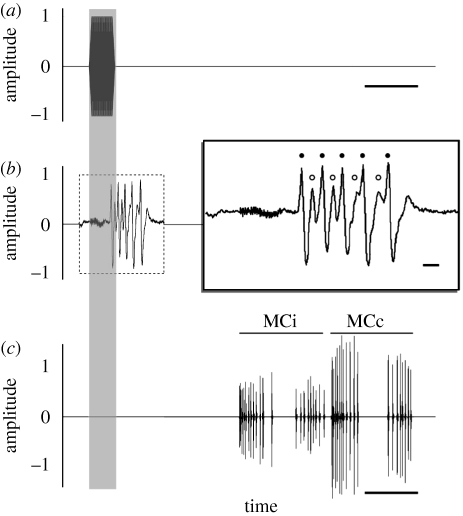
(*a*) A single 50 kHz, 5 ms pulse (0.5 ms rise/fall time inclusive; 95 dB peSPL) as used in the early-attack stimulus trains (figure 1). Scale bar, 10 ms. (*b*) Spike trace of auditory afferent activity in *C. tenera* to (*a*). Scale bar, 1 ms. Filled circle, A1; open circle, A2. (*c*) Phonoresponse of *C. tenera* to (*a*), illustrating ipsilateral (MCi) and contralateral (MCc) click modulation cycles. Scale bar, 10 ms. See [24] for a more detailed consideration of A1 and A2 cell spike sorting and the cyclical nature of tymbal activity. Note that in (*b*) the spike trace to the right is simply a magnification of the spike trace to the left which is, in turn, time-matched to (*a*,*c*) as indicated by the grey shadow spanning the three panels.

**Figure 3.**
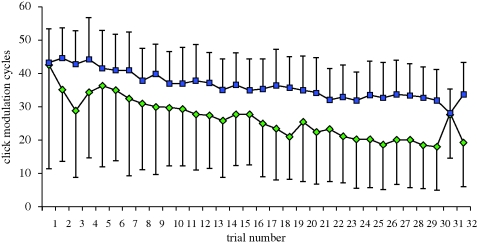
Top line (blue with squares, with upward standard deviation bars) shows the gradual pattern of habituation across trials 1 to 30 for the late-attack group, while the bottom line (green with diamonds, with downward standard deviation bars) illustrates the more profound pattern of habituation across trials 1 to 30 for the early-attack group. In response to early- and late-attack calls on trial 1, the two groups were equally responsive. On trial 31 individuals in both groups reacted with a number of MCs that was significantly different from the number produced during trial 30, indicating that the moths did not generalize between the two stimuli and, thus, discriminated between them. Conversely, neither group showed evidence of dishabituation to the original stimuli between trials 30 and 32.

Change at the sensory or motor level cannot explain the overall magnitude in the reduction in responsiveness across trials in either early- and late-attack groups because (i) within-trial sensory adaptation was not sustained across trials and (ii) the relatively strong response to late-attack calls on trial 31 in the early-attack group. This, then, argues against neural adaptation and muscle fatigue as primary contributors to this phenomenon but does not preclude the possibility that changes at the motor level, at least, have some influence (figures 2 and 3).

## 4. Discussion

Our results suggest that the dogbane tiger moth, *C. tenera* can discriminate between early- and late-approach phase calls of attacking bats, even when intensity and duty-cycle are held constant. Earlier, we found that *C. tenera* can identify different kinds of risk: the risk imposed by a bat in search of prey versus that imposed by one attacking [11]. However, this result may be attributable to differences in how moths encode bat echolocation calls arriving at their ears at rates above and below 10 Hz [1,10]. In the present study, we show that this same species is able to discriminate finer scale changes, between echolocation call repetition rates greater than or equal to 20 Hz, and do so based, presumably, on a different mechanism. We also found that *C. tenera* habituates more profoundly to sequences of early-approach phase pulses than to sequences of late-approach phase pulses, a difference which may reflect a simple but nevertheless adaptive specialization for iterative assessment and reassessment of tolerable risk and cost–benefit trade-offs over repeated predator encounters.

**Figure 4.**
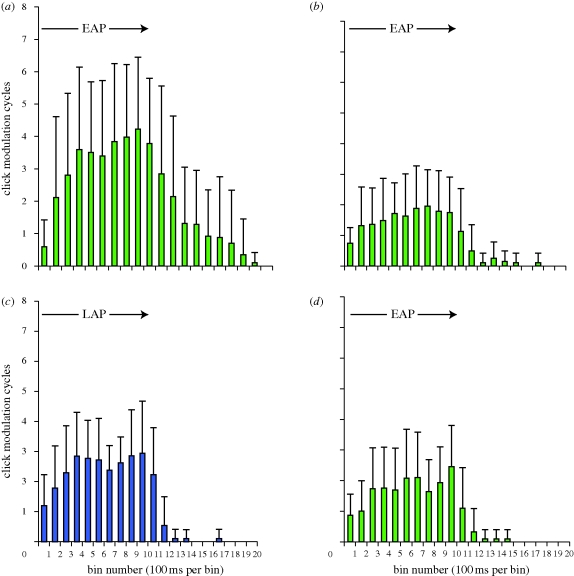
Average number of MCs produced by early-attack group on (*a*) trial 1, (*b*) trial 30, (*c*) trial 31 (presentation of late-attack stimulus train) and (*d*) trial 32. MCs that spanned two bins were divided according to the number of clicks that occurred in each of these two bins (i.e. a single MC that had 10 of its clicks fall in bin 1, and five of its clicks fall in bin 2 would have been assigned to these bins as 0.67 MCs (bin 1) and 0.33 MCs (bin 2)). Line and arrow indicate duration of stimulus presentation (EAP, early-attack presentation; LAP, late-attack presentation). Note that after the 10th bin most moths continued to produce sounds, that is, even after acoustic stimulation had ended.

We recently discovered that A1 auditory receptor cell activity alone is sufficient to elicit the phonoresponse in *C. tenera* and that the combined number of A1 and A2 spikes predicts the number of MCs that will be produced in response to a single pulse [24]. In the present study we control for stimulus intensity and duty cycle and show that the initial click phonoresponses to pulse trains simulating early- and late-approach phase calls are equivalent at the behavioural level. Neither of the repetition rates used was at the vertex (i.e. the repetition rate for which pulses of lowest relative intensity still elicit a phonoresponse [14]). Lack of stimulus generalization indicates that these insects are able to discriminate higher from lower risk predator cues. All else being equal, *C. tenera* consistently phonoresponds more vigorously to long duration pulses than to shorter duration pulses [11] and to single pulses of higher total energy (duration × intensity) than to single pulses of lower total energy [24]. Despite this, once habituated, moths in the lower risk group reacted to the higher risk stimulus (trial 31) by producing significantly more MCs than they had on trial 30 (figures 3 and 4). The opposite was true in the higher risk group (figure 3). Thus, pulse repetition rate, rather than pulse duration, is apparently the more salient of these two acoustic cues for assessing risk over repeated encounters (see also [11]).

*Cycnia tenera*'s phonoresponse habituated more rapidly to the lower risk stimulus than to the higher risk stimulus (figure 2). At equal pulse durations and intensities, more pulses per unit time predict more MCs [25]; however, controlling for pulse rate, pulses of lower total energy predict a lower phonoresponse relative to pulses of higher energy [11,24]. Our test of stimulus generalization shows that high- and low-risk pulse trains were discriminated, meaning that initially equivalent responses to these two stimuli must be due to balanced inequalities in pulse rate (lower for early-attack calls) and pulse duration (shorter for late-attack calls). Initial response equivalency of stimuli representing differing degrees of danger, at first glance, may not appear to be an optimal means of ‘first-pass’ risk assessment in nature. However, that *C. tenera* times its clicks to maximize their anti-bat defensive effects [13,26] helps explain the moth's peak sensitivity to bats calling at rates in between those used here. Initial stimuli equivalency on either side of this vertex may thus reflect design constraints of the underlying mechanism that has evolved, primarily, to react quickly to an attacking bat [27,28]. For initial risk-assessment, *C. tenera* appears to be dialled for sheer processing speed rather than accuracy (see [29] for review).

Eared moths detect, and are detected by, echolocating bats each night as they search for potential mates and oviposition sites [30,31]. Using an integrated discrimination test based on a combination of stimulus generalization and dishabituation, our study shows that *C. tenera* did not generalize between the two stimuli and is thus able to discriminate between attacking bats posing higher and lower levels of risk based on auditory cues. Conversely, a discrimination test based solely on a dishabituation assay would not have revealed this capability of *C. tenera*. The difference in this insect's sensitivity to these two tests may reflect an adaptive design. For predator risk-assessment there is no clear reason to why dishabituation would be beneficial after presentation of novel cue indicative of a different degree of danger. Conversely, the lack of stimulus generalization can be more easily explained. Between lower and then higher risk predator cues (simulating a bat that has just detected the moth versus one about to make contact), we see a marked increase in the number of bat-deterring defensive clicks produced, while a lack of stimulus generalization between higher and then lower risk predator cues (perhaps interpreted as a bat deterred and flying away) leads to a reduction in sound production, thereby minimizing the defence's energetic costs.

Our neural data show that there are more A1 and A2 spikes over the course of a late-attack trial (67, 1.5 ms pulses) than an early-attack trial (20, 5 ms pulses). In their seminal paper on the neural basis of habituation, Thompson & Spencer [22] predicted that greater overall neural activity at the sensory level would translate into faster rates of habituation. Our results do not directly support this. A lower rate of habituation was observed in response to the higher rate (late-attack) stimulus paradigm, although this paradigm evoked more auditory afferent activity than did the lower-rate (early attack) stimulus paradigm. However, individual late-attack pulses elicited fewer A cell spikes and longer spike periods than did individual early-attack pulses suggesting that, all else being equal, received pulse energy is a better predictor of habituation than pulse rate. Regardless, the slope describing habituation across trials was less steep in response to simulated late-attack calls than to simulated early-attack calls (figure 2), underscoring the importance of unidentified interneural mechanisms for balancing the competing interests of predator avoidance and mate finding in noctuoid moths [32].

*Cycnia tenera* lives for only a week or so as an adult, and is not known to replenish energy stores procured as a caterpillar. Iterative auditory risk assessment should allow *C. tenera* to decide how it will allocate time and resources, and suggests that over the course of repeated encounters with bats this moth does not react based solely on the activity of its four auditory afferents. Further research should reveal whether or not other moths and flying insects with simple bat-detecting ears (e.g. lacewings and mantises) possess functionally similar iterative risk-assessment mechanisms to balance the goal of reproduction and penalty of death.
